# Multi-omics analysis elucidates the relationship between intratumor microbiome and host immune heterogeneity in breast cancer

**DOI:** 10.1128/spectrum.04104-23

**Published:** 2024-03-05

**Authors:** Jia Li, Yu Zhang, Yifan Cai, Peizhuo Yao, Yiwei Jia, Xinyu Wei, Chong Du, Shuqun Zhang

**Affiliations:** 1 Department of Oncology, The Second Affiliated Hospital of Xi'an Jiaotong University, Xi’an, Shaanxi, China; University of Arkansas for Medical Sciences, Little Rock, Arkansas, USA

**Keywords:** intratumor microbiome, breast cancer, tumor immune microenvironment, immunotherapy, prognostic factors

## Abstract

Research has indicated that intratumor microbiomes affect the occurrence, progression, and therapeutic response in many cancer types by influencing the immune system. We aim to evaluate the characteristics of immune-related intratumor microbiomes (IRIMs) in breast cancer (BC) and search for potential prognosis prediction factors and treatment targets. The clinical information, microbiome data, transcriptomics data of The Cancer Genome Atlas Breast Invasive Carcinoma (TCGA-BRCA) patients were obtained from Kraken-TCGA-Raw-Data and TCGA portal. The core tumor-infiltrating immune cell was identified using univariate Cox regression analysis. Based on consensus clustering analysis, BC patients were categorized into two immune subtypes, referred to as immune-enriched and immune-deficient subtypes. The immune-enriched subtype, characterized by higher levels of immune infiltration of CD8+ T and macrophage M1 cells, demonstrated a more favorable prognosis. Furthermore, significant differences in alpha-diversity and beta-diversity were observed between the two immune subtypes, and the least discriminant analysis effect size method identified 33 types of IRIMs. An intratumor microbiome-based prognostic signature consisting of four prognostic IRIMs (*Acidibacillus*, *Succinimonas*, *Lachnoclostridium*, and *Pseudogulbenkiania*) was constructed using the Cox proportional-hazard model, and it had great prognostic value. The prognostic IRIMs were correlated with immune gene expression and the sensitivity of chemotherapy drugs, specifically tamoxifen and docetaxel. In conclusion, our research has successfully identified two distinct immune subtypes in BC, which exhibit contrasting prognoses and possess unique epigenetic and intratumor microbiomes. The critical IRIMs were correlated with prognosis, tumor-infiltrating immune cell abundance, and immunotherapeutic efficacy in BC. Consequently, this study has identified potential IRIMs as biomarkers, providing a novel therapeutic approach for treating BC.

**IMPORTANCE:**

Recent research has substantiated the presence of the intratumor microbiome in tumor immune microenvironment, which could influence tumor occurrence and progression, as well as provide new opportunities for cancer diagnosis and treatment. This study identified the critical immune-related intratumor microbiome (*Acidibacillus*, *Succinimonas*, *Lachnoclostridium*, and *Pseudogulbenkiania*), which were correlated with prognosis, tumor-infiltrating immune cell abundance, and immunotherapeutic efficacy in breast cancer and might be the novel target to regulate immunotherapy in BC.

## INTRODUCTION

Breast cancer (BC) is the most common cancer among women, comprising 31% (297,790/948,000) of new cases and 15% (43,170/287,740) of projected fatalities of women’s cancer in 2023 (1). Despite advancements in treatment, BC patients experience unfavorable outcomes due to recurrence, metastasis, and resistance (2). So, there is an urgent need to identify prognostic and therapeutic factors associated with BC patients to develop novel treatment and diagnostic approaches.

The tumor microenvironment (TME) in solid tumors is highly complex (including tumor, immune, and stroma cells interacting with the extracellular matrix). The TME exhibits variations across different races and cancer subtypes, strongly affecting the clinical outcomes and therapeutic responses (3–5). The tumor immune microenvironment (TIME) specifically refers to the immune component within the tumor, which significantly influences tumor progression and anticancer treatments (6). For instance, an upregulation of CD4 and CD8-positive T cells is related to the favorable prognosis in triple-negative breast cancer (TNBC) (7), while the presence of CD25^high^ effector regulatory T cells hampers responses to programmed cell death protein 1 (PD-1) blockade in TNBC (8). Considering the complexity and significance of TIME, it is crucial to investigate the variations in immune microenvironment characteristics for disease classification and precise targeted therapy. In BC, numerous immune subtypes have been developed to aid in the identification, prognosis prediction, and treatment guidance, such as clusters based on profiling of single T helper (TH) cells (including TH-activated and TH-silenced clusters) (9) and three 22 immune cell-based infiltration patterns (including immune-excluded, immune-desert, and immune-inflamed phenotypes) (10). However, it is imperative to acknowledge that not all immune cells possess substantial relevance in the TME or the development of tumors. Therefore, it is crucial to identify immune cell types of greater significance for further investigation. Previous research has demonstrated that the infiltration patterns of core immune cells can identify molecular subtypes and enhance the efficacy of targeted immunotherapy in cutaneous melanoma (11). In light of the circumstances above, it is essential to optimize existing predictive models and comprehensively assess the presence of functional immune cells related to the prognosis of BC patients, thereby reflecting the overall state of the TME.

Recent research has substantiated the presence of the intratumor microbiome (IM) in TIME, which has the potential influence on tumor occurrence and progression (12–14), as well as provides new opportunities for cancer diagnosis and treatment (15). BC exhibits a more diverse microbiome than normal breast samples and other types of cancer, with variations observed in race, stage, and BC subtypes (12, 16). Studies indicated that microorganisms can participate in cancer cell metastasis and lung colonization (17), correlating with prognostic features and immunological signatures in BC (18). Furthermore, phenotypically specific intratumor microbiomes could contribute to tumor biomarkers and treatment innovation. Metabolism-related microbiomes can predict the prognosis of BC patients (19). The tumor microbiome could independently predict ovarian cancer prognosis and interact with immune genes and cells (20). It is worth noting that regulating the microbiome might provide new ways to enhance the effectiveness of immunotherapy. Fungal mycobiome could stimulate pancreatic tumor growth by driving interleukin (IL)-33 secretion and type 2 immunity; thus, anti-fungal treatment might benefit pancreatic tumors (21). Dietary tryptophan metabolite released by intratumor *Lactobacillus reuteri* could facilitate immune checkpoint inhibitor (ICI) treatment in melanoma (22). Therefore, identifying the core immune characteristics in BC and exploring the impact of the microbiome on the TIME will help further understand the difference in tumor immunotherapy and actively facilitate the development of targeted microbiome therapies to enhance the prognosis of BC patients. Whole-transcriptome analysis with total RNA sequencing and intratumor microbiome data provided by The Cancer Genome Atlas (TCGA) present a valuable approach for examining the relationship between IM and host gene expression (20, 23, 24).

This study used a multigroup approach to evaluate the TIME pattern of BC patients and investigate the association between immune status and prognosis, epigenetic, and intratumor microbiome characteristics, aiming to elucidate their influence on the establishment and maintenance of the TIME. By identifying immune-related intratumor microbiome (IRIM) biomarkers of BC, an intratumor microbiome prognostic signature was developed, enabling the prediction of clinical prognosis, response to immunotherapy, and selection of chemotherapy drugs for patients.

## MATERIALS AND METHODS

### Data collection

The RNA sequencing data and corresponding clinical profiles were acquired from the TCGA database (https://tcga-data.nci.nih.gov/tcga/). The abundances of immune cell data of the TCGA-BRCA samples were downloaded from Immune Cell Abundance Identifier (ImmuCellAI) (http://bioinfo.life.hust.edu.cn/ImmuCellAI#!/) (25) and CIBERSORTx (https://cibersortx.stanford.edu/index.php) (26). The 24 immune cell types, including 18 T‐cell subsets (CD4+, CD8+, CD4+ naïve, CD8+ naïve, central memory T, effector memory T, Tr1, iTreg, nTreg, Th1, Th2, Th17, Tfh, Tc, MAIT, Tex, gamma delta T, and natural killer T cells) and six other important immune cells (B cells, macrophages, monocytes, neutrophils, dendritic cell (DC), and natural killer (NK) cells), were identified by the marker genes in the ImmuCellAI (Table S1) (25). For metagenomic information, Kraken-TCGA-Raw-Data (*n* = 17,625) and Metadata-TCGA-Kraken-17625-Samples were obtained from the repository (ftp://ftp.microbio.me/pub/cancer_microbiome_analysis/) (27). We remained 1,018 TCGA-BRCA samples with overall survival (OS) more than 1 month and primary BC (TCGA sample code 01) for this study (Table S2).

### Utilization of consensus clustering to identify immune subtypes based on core tumor-infiltrating immune cells (cTICs)

Univariate Cox analysis was used to determine the cTICs (*P* value < 0.05) among all 24 tumor immune cells in BC. In order to further elucidate the biological significance of cTICs in BRCA, the “ConsensusClusterPlus” R package (28) was employed to categorize the BRCA samples into distinct subgroups based on the cTICs. The unsupervised clustering method “k-means” based on a Euclidean distance metric and Ward’s linkage was utilized in this analysis, with 1,000 repetitions to ensure the stability of the classification.

### Immune landscape analysis between the immune subtypes

The ESTIMATE algorithm could evaluate the fraction of stromal and immune cells based on gene expression signatures, which could provide three immune-related enrichment scores: stromal, immune, and ESTIMATE (29). Furthermore, to evaluate the predicted value of the immune subtypes in immunotherapy, we analyzed the expression of the immune checkpoints (ICPs). The immunophenotype scores (IPS) could measure patient response to anti-cytotoxic T lymphocyte associate protein-4 (CTLA-4) and anti-PD-1 therapy (30). Tumor Immune Dysfunction and Exclusion (TIDE, http://tide.dfci.harvard.edu/) integrates the expression signatures of T cell dysfunction and T cell exclusion to model tumor immune evasion (31, 32). TIDE prediction scores represent the potential of tumor immune escape and can evaluate the intrinsic ICI resistance.

### Intratumor microbiome analysis between the immune subtypes

The alpha-diversity [measured by abundance coverage-based estimator (ACE), Chao 1, and observed indexes] and beta-diversity [measured by principal coordinate analysis (PCoA) based on Bray-Curtis distance] were analyzed in two immune subtypes using vegan (v2.6-4) R package (33). To identify intratumor microbiomes that significantly differ as immune-related biomarkers between the two immune subtypes, we conducted a Linear discriminant analysis Effect Size (LEfSe) analysis at the order, family, and genus taxonomic levels (34).

### Joint analysis of IRIMs and Construction of intratumor microbiome-based prognostic signature (IMBPS)

Heatmaps of the matrix of Pearson correlations visualize the correlations between infiltrating immune cells and IRIMs and the correlations between TIME characteristics and IRIMs by “pheatmap” (v1.0.12) R package (35). A correlation network composed of IRIMs and genes associated with immunity was constructed. Data for 148 immunomodulators and inhibitory immune checkpoints were collected from previous studies (36). Pearson correlation coefficients and *P*-values were calculated with the “Hmisc” R package (v4.7.1) (37), and the significant correlations (*P* < 0.05) were visualized by the Cytoscape (v3.9.0) (38). The IMBPS were constructed with the LASSO Cox regression analysis (LASSO, least absolute shrinkage, and selection operator) and multivariate Cox regression analysis.

### Function enrichment analyses and aberrant pathways between different groups

Gene set variation analysis (GSVA) was performed to analyze the differences in biological function between the different groups with the “GSVA” R package (39). Gene set enrichment analysis (GSEA) was used to analyze the potential signaling pathway related to the IRIMs (40). The gene sets “h.all.v2022.1.Hs.symbols” and “c2.cp.kegg.v7.5.1.symbols” were obtained from the MSigDB database to run GSVA and GSEA. “PROGENy” R package (41) was performed to infer 14 signaling and regulatory pathways, including androgen, epidermal growth factor receptor (EGFR), estrogen, hypoxia, JAK-STAT, MAPK, NF-kB, p53, PI3K, TGF-β, TNF-α, Trail, VEGF, and WNT.

### Drug sensitivity evaluation

Drug sensitivity was estimated using the “pRRophetic” R package (42). We compared the half maximal inhibitory concentration (IC50) values in the two groups and explored the associations between the IC50 values, IMRPS score, and the four IM levels.

### Statistical analysis

The statistical analysis was conducted using the R software (version 4.0.5). The Wilcoxon signed-rank test was employed to compare the differences between the two groups. All tests were two-sided, with a *P*-value of less than 0.05, indicating statistical significance. The significance levels were set at *P ≤ 0.05, **P ≤ 0.01, and ***P ≤ 0.001.

## RESULTS

### Identification of immune subtypes based on cTICs

**Fig 1:**
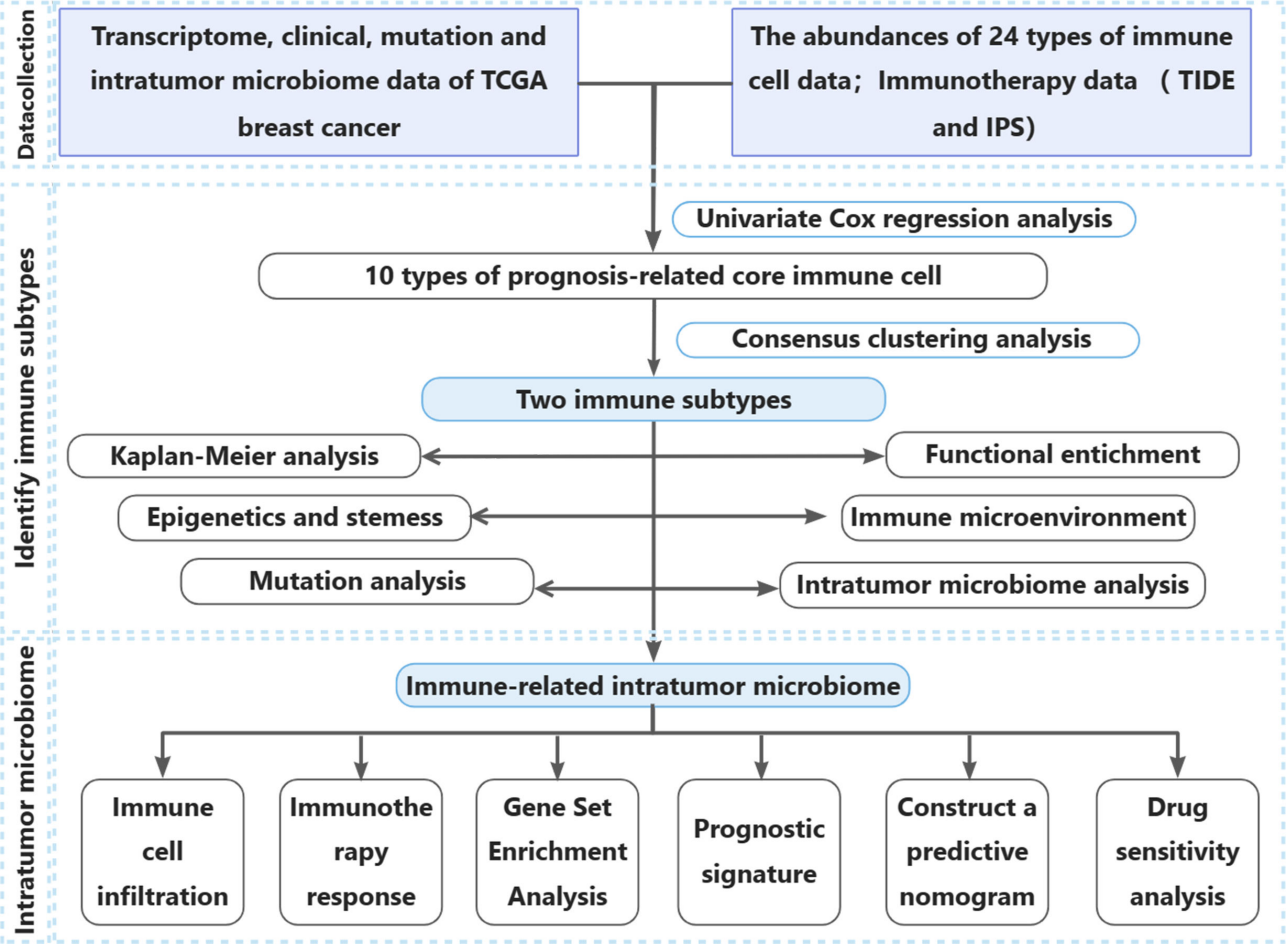
Study flow diagram.

**Fig 2:**
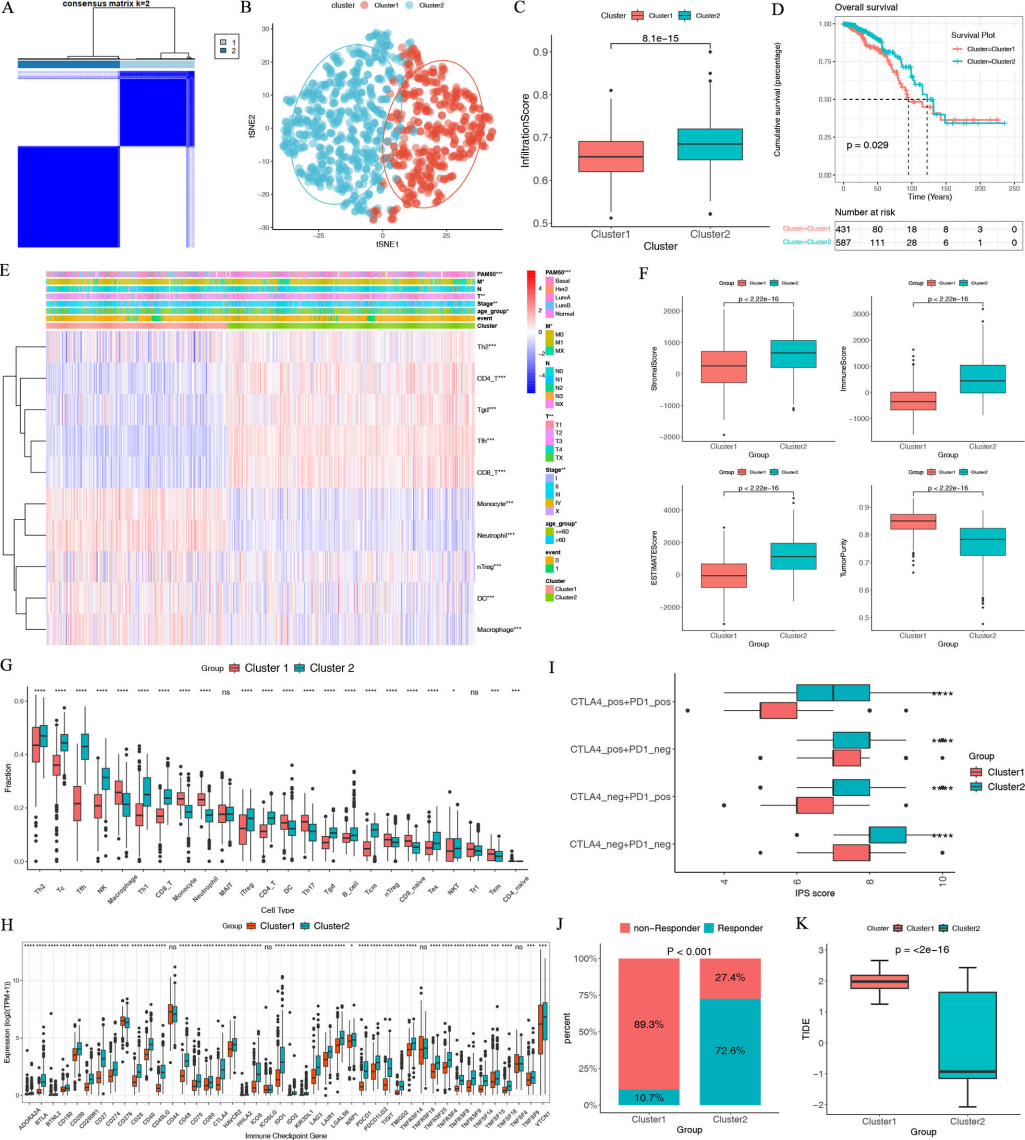
Two breast cancer microenvironment subtypes were revealed by unsupervised analysis of the cTIC. (**A**) The consensus matrix when *k* = 2. (**B**) The t-Distributed Stochastic Neighbor Embedding (t-SNE) plots of the two clusters. (**C**) Comparison of the infiltration score between two clusters. (**D**) Kaplan-Meier (KM)curves of the two clusters. (**E**) Heat map of immune cells in TCGA-BRCA cohorts. (**F**) Comparison of the immune, stromal, and estimated scores and tumor purity between two clusters. (**G**) Comparisons of the infiltration fractions of immune cells according to the ImmuCellAI between two clusters. (**H**) Comparisons of the ICGs between two clusters. (**I**) Comparisons of the IPS scores between two clusters. (**J**) Comparisons of the non-responders and responders to immunotherapy according to the TIDE algorithm between two clusters. (**K**) Comparisons of the TIDE scores between two clusters.

The study flow diagram is shown in Fig. 1. Univariate Cox analysis identified 10 cTICs, including activated memory T helper 2 (Th2), CD4+ T, CD8+ T, tgd, tfh, monocyte, neutrophil, nTreg, DC, and macrophage cells (Table S3). Two immune subtypes were identified with unsupervised clustering based on the 10 cTICs, termed Cluster 1 and Cluster 2 (Fig. 2A and B). Infiltration scores significantly differed between the two independent immune subtypes (Fig. 2C). The prognosis of patients in Cluster 2 was better than Cluster 1 (Fig. 2D). Figure 2E displayed the clinical pathological parameters between the two clusters. Patients in Cluster 2 had higher stromal and immune scores and lower tumor purity (Fig. 2F). The stromal and immune scores could predict the level of infiltrating stromal and immune cells and these form the basis for the ESTIMATE score to infer tumor purity in tumor tissue. The proportion of macrophage M0 was higher in Cluster 1, while the proportions of CD8+ T cells, activated CD4+ memory T cells, and macrophage M1 were significantly higher in Cluster 2 (Fig. 2G; Fig. S1). Immune checkpoint genes (ICGs) are also associated with immunotherapy effectiveness (43), and patients with high immunotherapy effectiveness may express more ICGs (44). We found a greater level of expression of ICGs in Cluster 2 than in Cluster 1, which indicates that Cluster 2 might respond better to immunotherapy (Fig. 2H). A comparison of the IPS and TIDE scores between Cluster 1 and Cluster 2 was conducted to examine the treatment response predicting the significance of the clusters. IPS was developed from four classes immune-related genes, including effector cells, immunosuppressive cells, major histocompatibility complex (MHC) molecules, and selected immunomodulatory. The IPS score has predictive value in patients treated with the CTLA-4 and PD-1 blockers. The higher the IPS score was, the more potential there was to respond to CTLA-4 and PD-1 blockers. IPS scores (ips_ctla4_neg_pd1_neg, ips_ctla4_neg_pd1_pos, ips_ctla4_pos_pd1_neg, and ips_ctla4_pos_pd1_pos) were higher in Cluster 2 (Fig. 2I), indicating that Cluster 2 tended to have better immunotherapy response. Cluster 2 had more responders than Cluster 1 and had a lower TIDE score (Fig. 2J and K). An increased TIDE score is related to immune escape. Thus, we identified two different immune subtypes. Cluster 1 with a longer OS has a better response to ICI than the non-inflamed Cluster 2.

### Differences in epigenetic and genetic factors between the immune subtypes

**Fig 3:**
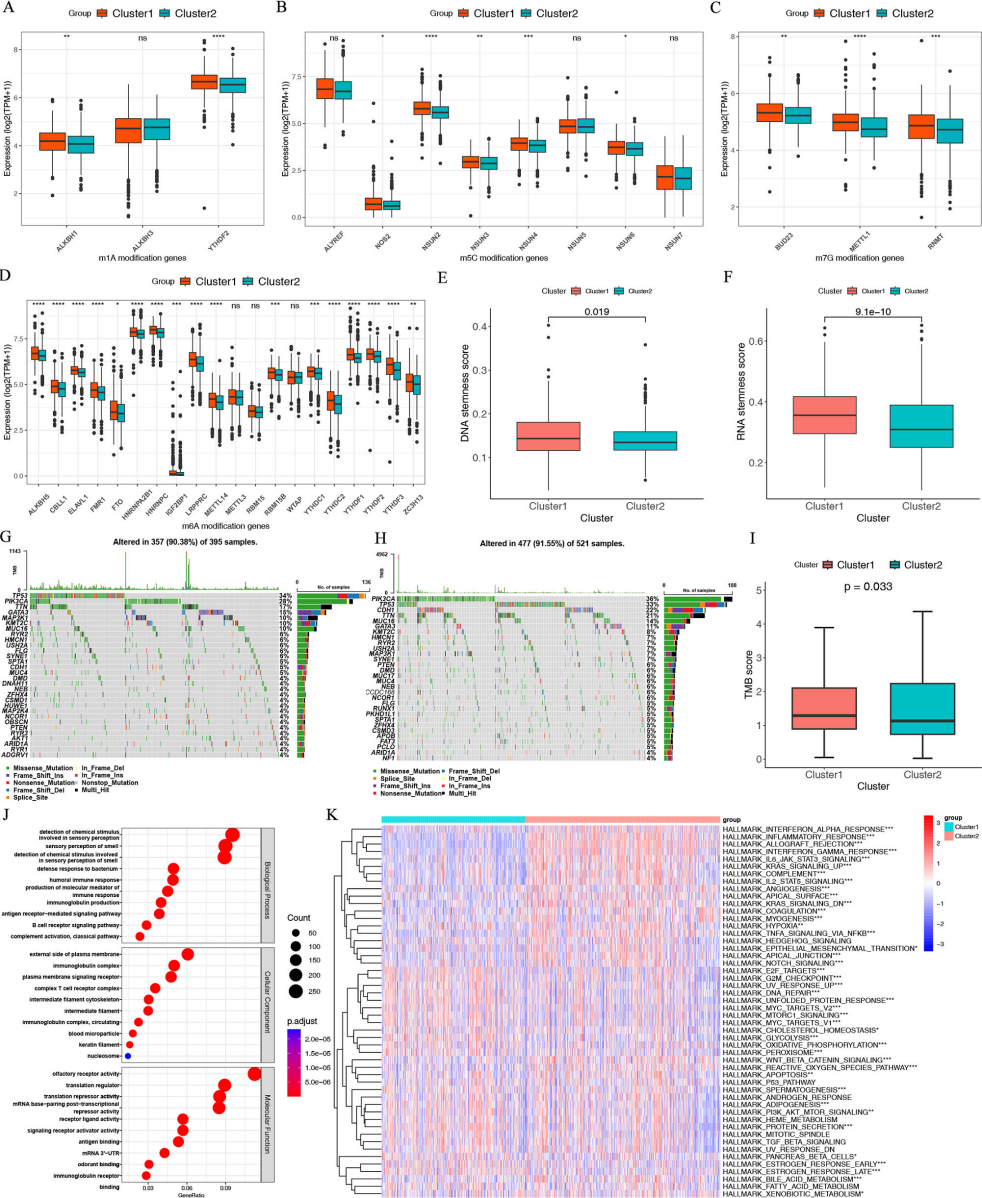
Differences in epigenetics, gene mutation, and functional pathways between the immune subtypes. Differences in the expression of (**A**) m1A, (**B**) m5C, (**C**) m7G, and (**D**) m6A regulatory molecules between the two immune subtypes. Differences in (**E**) DNA stemness score (DNAss) and (**F**) RNA stemness score (RNAss) between the immune subtypes. Overview of mutation frequency and type in (**G**) Cluster 1 and (**H**) Cluster 2. (**I**) Differences in tumor mutation burden (TMB) score between the two immune subtypes. (**J**) Gene Ontology (GO) enrichment analysis results of the differentially expressed genes between the immune subtypes. (**H**) GSVA for HALLMARK pathways analysis.

In order to understand the factors that affect the difference in TME between two immune subtypes, we explored three aspects: epigenetics, gene mutation, and functional pathways. RNA methylation could regulate the innate immunity of the body, and targeting these methylation regulatory molecules (such as METTL3, METTL4, and FTO) can directly enhance tumor immunotherapy (45). We examined the four types of RNA methylation regulatory molecules, including N6-methyladenosine (m6A), 5-methylcytosine (m5C), N1-methyladenosine (m1A), and N7-methylguanosine (m7G). We found a significant increase in RNA methylation regulatory molecules in Cluster 1 with poor prognosis (Fig. 3A through D).

Additionally, tumor stemness can influence the immune characteristics of cancer, such as the ICPs and the immune cells (46). To assess tumor stemness, RNA stemness score (RNAss) and DNA stemness score (DNAss) can be calculated based on mRNA expression levels (46). The cancer RNAss and DNAss were lower in Cluster 2 (Fig. 3E and F). Figure 3G and H visualized the top 20 genes with the highest alteration frequency and showed that Cluster 2 had a higher overall gene mutation frequency than Cluster 1. The Cluster 2 subtype harbored a higher tumor mutation burden (TMB) score than Cluster 1 (Fig. 3I). Previous research has shown that a high TMB is linked with higher numbers of potentially immunogenic neoantigens (47) and the activation of infiltrating CD8+ T cells that may facilitate anti-tumor immune responses (48).

Enrichment analysis revealed functional differences between the two immune subtypes. GO enrichment analysis indicated that the differential genes were enriched in bacterial response (defense response to bacterium) and immune-related pathways (humoral immune response, immunoglobulin production, and B cell receptor signaling pathway) (Fig. 3J). The GSVA indicated that processes associated with more aggressive cancer were activated in Cluster 1, while processes associated with more activated immunity were enriched in Cluster 2 (Fig. 3K). The consistency among the epigenetics, gene mutation, functional pathways, and immune subtypes might indicate that the classification of immune subgroups is reasonable.

### Identification of the IRIMs

**Fig 4:**
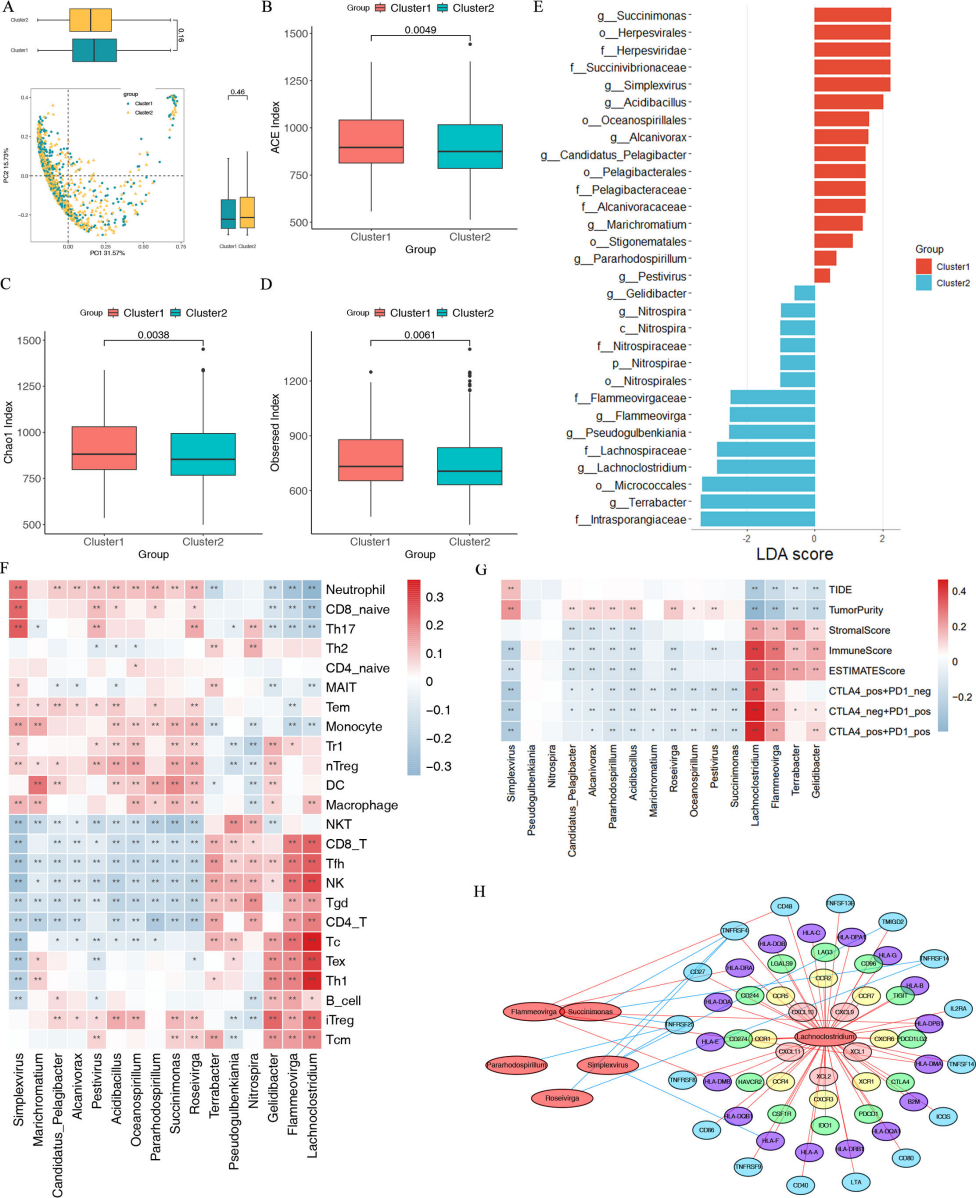
Cluster 1 and Cluster 2 exhibited different intratumor microbiome profiles. (**A**) PCoA and box plot shown along the first two principal coordinates on the Bray-Curtis distances for likely microbiome composition data. Comparison of alpha-diversity (**B**) ACE index, (**C**) Chao 1 index, and (**D**) observed index in the two immune subtypes. (**E**) Significant differentially abundant taxonomic biomarkers between Cluster 1 and Cluster 2 identified by LEfSe. (**F**) Correlation between microbiome abundance and immune cell components. (**G**) Correlation between microbiome abundance and TIDE, ESTIMATE, and IPS scores. (**H**) Association between immunomodulatory genes and microbes. Associations with Spearman correlation coefficients of >0.3 and *P*-value of <0.05 are shown.

Intratumor microbiomes were found to be closely related to cancer immunity. Since enrichment analysis revealed significant differences in the defense response to bacterium activity between the two clusters, we further explored the distribution and characteristics of intratumor microbiomes between the two subtypes. We use alpha-diversity and beta-diversity to reveal the differences between the immune subtypes. Although beta-diversity, which is defined as the dissimilarity among communities, showed no significant difference between the two immune subtypes (*P* > 0.05) (Fig. 4A), alpha-diversity analysis showed that intratumor microbiome profiles were different between Cluster 1 and Cluster 2 (*P* < 0.05) (Fig. 4B and D). The LEfSe and least discriminant analysis effect size indicated 33 types of dominant bacterial community differences between the two immune subtypes (Table S4). Figure 4E shows the two immune subtypes’ top 30 significant microbial biomarkers.

In order to better understand the relationship between the above IRIMs and BC’s TIME, we analyzed the correlations among the intratumor microbiomes and tumor-infiltrating immune cells (TIICs). The Spearman correlation analysis found that the intratumor microbiomes significantly correlated with 24 immune cell types (Fig. 4F). For example, as the biomarker of the Cluster 2 subtype and the predictor of good survival, *Flammeovirga, Lachnoclostridium, Terrabacter, Gelidibacter, Pseudogulbenkiania,* and *Nitrospira* were positively correlated with the anti-tumor immune cells, including NK cells and CD4 and CD8 cells, but negatively associated with CD8 naïve cells and monocyte cells. As the biomarker of the Cluster 1 subtype and the predictor of poor survival, the *Simplexvirus, Pestivirus, Marichromatium, Pararhodospirillum, Acidibacillus, Candidatus Pelagibacter, Alcanivorax, Succinimonas, Oceanospirillum,* and *Roseivirga* were negatively correlated with the anti-tumor immune cells, including NK cells and CD4 and CD8 cells. The above analysis shows that we have identified the immune-related intratumor microbiomes that might play an essential role in BC.

### Exploration of the relationship between 16 IRIMs and TIME and ICI therapeutic response

Immunogenicity of microorganisms can trigger the immune system of the host, thereby increasing the effectiveness of immune checkpoint inhibitors (49). Therefore, the intratumor microbiome is essential in promoting or suppressing cancer and affects the overall anti-tumor immunotherapy effect (21). We evaluated the correlation between 16 IRIMs at the genus level and immune efficacy prediction indicators. *Flammeovirga, Lachnoclostridium, Terrabacter,* and *Gelidibacter* had positive correlations with the ESTIMATE, stromal, and immune scores, indicating that they have an active anti-tumor immune microenvironment (Fig. 4G). At the same time, these microbes also positively correlated with ips_ctla4_neg_pd1_pos (CTLA-4-negative response and PD-1-positive response), ips_ctla4_pos_pd1_neg, and ips_ctla4_pos_pd1_pos. In contrast, these IRIMs were negatively correlated with tumor purity and TIDE score, which indicated more tumor malignancy and unfavorable immune-treatment response (Fig. 4G). As for the IRIMs enriched in Cluster 1 (*Simplexvirus, Pestivirus, Marichromatium, Pararhodospirillum, Acidibacillus, Candidatus_Pelagibacter, Alcanivorax, Succinimonas, Oceanospirillum,* and *Roseivirga*), they had negative correlations with ESTIMATE score, stromal score, immune score, ips_ctla4_neg_pd1_pos, ips_ctla4_pos_pd1_neg, and ips_ctla4_pos_pd1_pos. However, they were positively correlated with tumor purity and TIDE score (Fig. 4G). However, *Pseudogulbenkiania* and *Nitrospira* had no associations with the above indicators (Fig. 4G). Our analysis indicates a correlation between 16 IRIMs, immune cell infiltration, and the immune efficacy prediction indicators. There was a potential that the IRIMs would be able to predict TIME and immunotherapy response in BC.

### Microbiomes might affect TIICs by regulating the expression of immune-related genes

Research has shown that the intratumor microbiome can modulate immune-related genes and infiltrate immune cells directly or indirectly (50, 51). We further analyzed the associations between the IRIMs and 148 immune-related genes, and six IRIMs (*Lachnoclostridium, Succinimonas, Flammeovirga, Pararhodospirillum, Simplexvirus,* and *Roseivirga*) were associated with at least one immune-related gene (Fig. 4H). The *Lachnoclostridium* was positively correlated with many immune-related genes, such as TNFRSF4, HLA-F, CD27, PDCD1, CTLA-4, PDCD1LG2, TIGIT, and LAG3. The *Flammeovirga* had positive correlations with CD48, HLA-DRA, HLA-DPB1, HLA-DMA, and HLA-DMB. The *Succinimonas* was negatively correlated with TNFRSF4, TNFRSF14, and TNFRSF25. The *Pararhodospirillum* had negative correlations with TNFRSF25. The *Simplexvirus* had negative correlations with TNFRSF25, TNFRSF4, TMIGD2, and HLA-F. The *Roseivirga* had negative correlations with TNFRSF4 and TNFRSF14. These results indicated that the IRIMs might influence antigen processing and anti-tumor immunity by regulating the immune-related genes.

### Construction of IMBPS and nomogram

**Fig 5:**
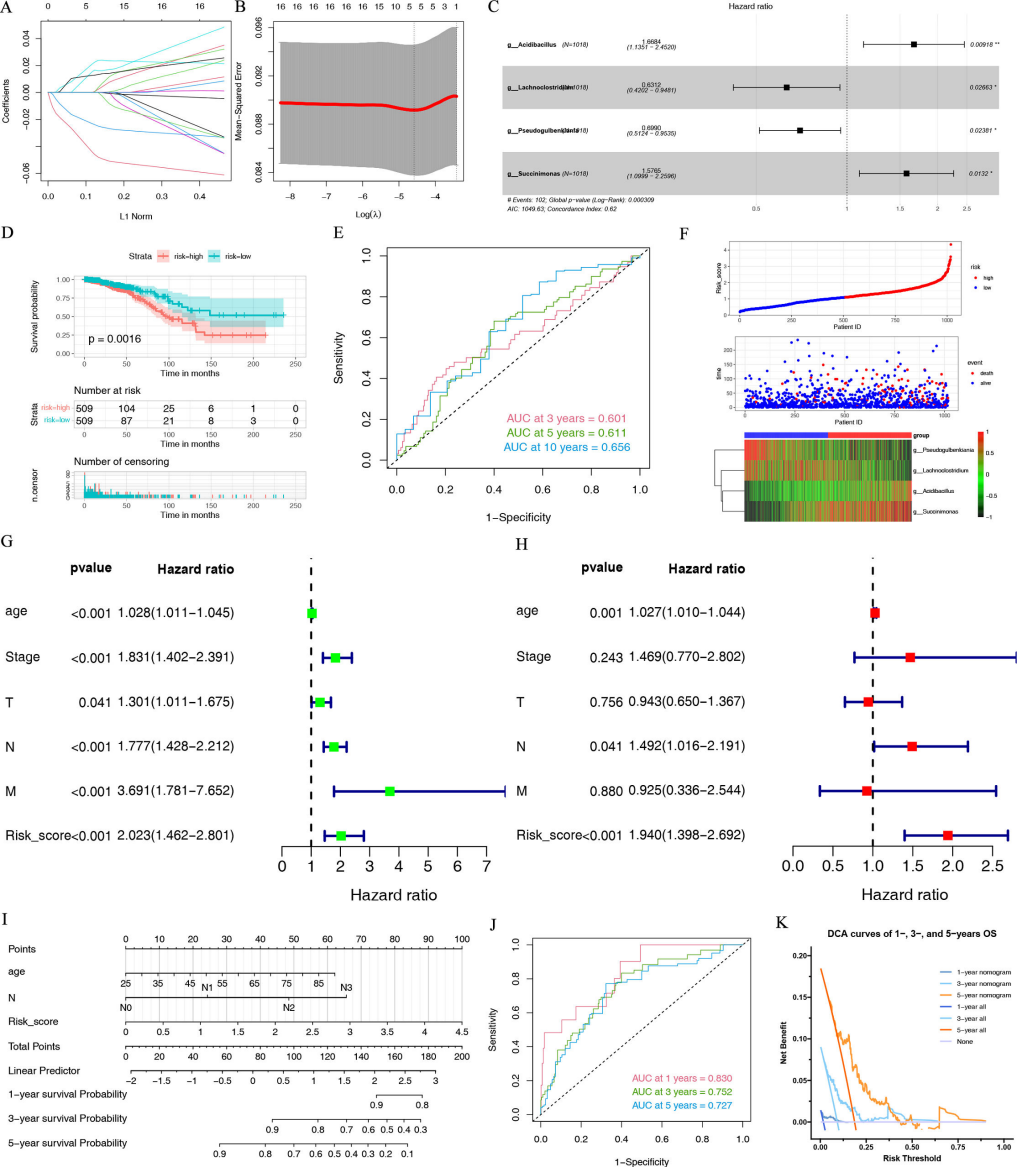
Construction of the intratumor microbiome-related prognostic signature. (**A**) LASSO regression analysis of 108 prognostic differentially expressed genes (DEGs). (**B**) Cross validation method to select optimal genes. (**C**) A forest plot shows the results of the multivariate Cox regression analysis. (**D**) KM curves of the OS. (**E**) Three-, 5-, and 10-year receiver operating characteristic (ROC) curves of the IMBPS. (**F**) The risk distribution plot in the TCGA cohort. The univariate (**G**) and multivariate (**H**) Cox regression analyses in the TCGA cohort. (**I**) The nomogram for predicting the 1-, 3-, and 5-year OS probabilities. (**J**) ROC curves of the nomogram. (**K**) The nomogram’s 1-, 3-, and 5-year decision curve analysis (DCA) curves indicated its net clinical benefits.

Intratumor microbiomes play a prognostic role in many cancers. Univariate Cox proportional-hazard model analysis found that the intratumor microbiomes could be the BC patients’ OS, disease-specific survival, progression-free survival, and disease-free survival prognosis biomarkers (Tables S5 to S8). The IMBPS consisted of two protective IRIMs (*Acidibacillus* and *Succinimonas*) and two risk IRIMs (*Lachnoclostridium* and *Pseudogulbenkiania*) (Fig. 5A through C). According to the median IMBPS score, The BC patients were divided into high- and low-risk groups. The survival analysis indicated that the prognosis in the low-risk group was better (Fig. 5D). The area under the curves (AUCs) of IMBPS score in predicting the 3-, 5-, and 10-year OS were 0.601, 0.611, and 0.656 (Fig. 5E). In general, the test with the higher AUC may be considered better, and an AUC of 0.6–0.7 is considered acceptable whereas an AUC >0.7 is considered good (52). The distributions of the risk score, survival outcomes, and four intratumor microbiome expressions are shown in Fig. 5F. Figure 5G and H indicated that the IMBPS score was the independent prognostic factor (*P* < 0.05). Furthermore, the three independent prognostic factors established the nomogram to quantitatively estimate the BC patients’ OS (Fig. 5I). Figure 5J and K demonstrate that the nomogram has high accuracy and can provide clinical benefits. The above results indicate that the IRIMs-based prognostic signature and nomogram could predict the BC patients’ prognosis and assist the clinical management.

### Molecular and immune characteristics of different IMBPS risk groups

**Fig 6:**
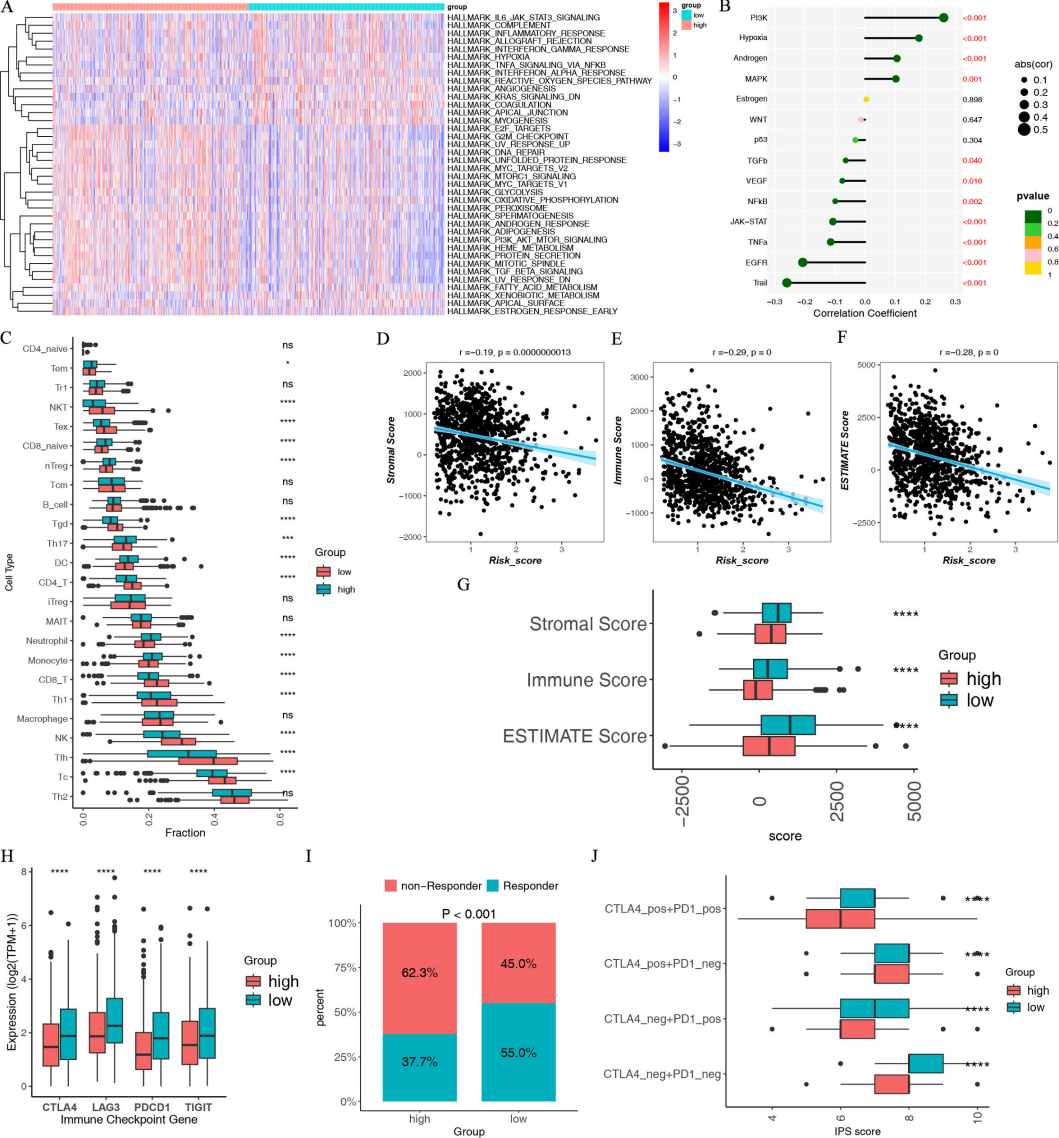
Molecular and immune characteristics of different IMBPS risk groups. (**A**) GSVA for HALLMARK pathways analysis. (**B**) Correlation between pathway activity estimated by PROGENy algorithm and immune-related microbial score. (**C**) Differences in infiltration fractions of 24 immune cell subsets between the two risk groups according to the ImmuCellAI database. Correlation between the (**D**) stromal, (**E**) immune, and (**F**) estimated scores with the immune-related microbial score. (**G**) Differences in immune, stromal, and estimated scores between the two risk groups. (**H**) Differences in ICPs between the two risk groups. (**I**) Differences in immune, stromal, and estimated scores between the two risk groups. (**J**) Differences in the IPS between the two risk groups stratified by CTLA-4 and PD-1.

We conducted the functional enriched analysis to uncover the characteristics of the IMBPS risk groups. The GSVA revealed that the low-risk group exhibited enriched immune-related pathways (such as the IL6-JAK-STAT3 signaling and inflammatory response pathways), while the high-risk group exhibited enriched cancer-related pathways (such as PI3K-AKT-MTOR signaling and G2M checkpoint pathways) (Fig. 6A). PROGENy analysis indicated that the IMBPS score had a positive association with oncogenic driver (mainly PI3K, hypoxia, and MAPK) pathways, while the IMBPS score had a negative association with the cell death (Trail) pathway (Fig. 6B).

Furthermore, we analyzed other vital immune features. Many anti-tumor lymphocyte cell subpopulations were much higher in the low-risk group, such as T cell subsets, B cells, and NK cells, while macrophage M2 was higher in the high-risk group (Fig. 6C). The ESTIMATE results indicate that the immune, stromal, and ESTIMATE scores were increased in the low-risk group and had negative associations with the IMBPS risk score (Fig. 6D through F). Strikingly, the IMBPS score had negative correlations with the immune, stroma, and ESTIMATE scores (Fig. 6G).

Blocking immune checkpoint pathways is an approach to anticancer immunity, and increased expression of ICPs is related to better responses to ICI (53). Four common ICPs were expressed at higher levels in the low-risk group and negatively correlated with the IMBPS score (Fig. 6H). We further assessed the immunotherapy response according to TIDE and IPS. IPS scores and responders to immunotherapy were higher in the low-risk group, suggesting they could benefit more from immunotherapy (Fig. 6I and J). Based on these findings, ICI treatment would benefit low-risk patients more than high-risk patients.

### Microbiome abundance correlation to oncogenic and immune pathways

**Fig 7:**
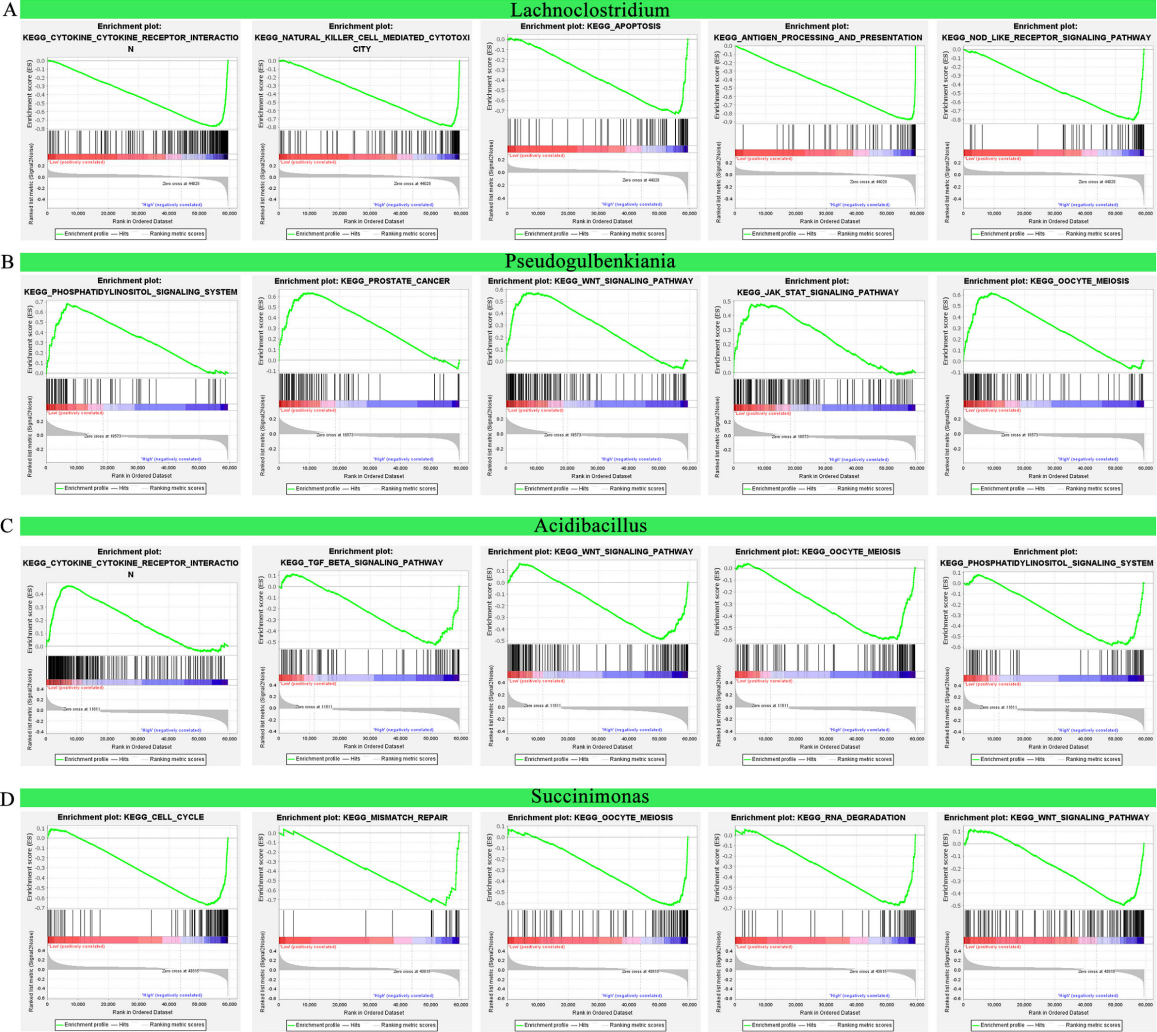
Microbiome abundance correlation to oncogenic and immune pathways. The (**A**) *Lachnoclostridium*, (**B**) *Pseudogulbenkiania*, (**C**) *Acidibacillus*, and (**D**) *Succinimonas*-related oncogenic and immune pathways estimated by GSEA.

Microbiomes can regulate the immune through many pathways. GSEA was used to explore the possible pathways associated with IRIMs. A high abundance of *Lachnoclostridium* was related to immune-related pathways, such as antigen processing and presentation, apoptosis, cytokine-cytokine receptor interaction, natural killer cell-mediated cytotoxicity, and nucleotide-binding oligomerization domain (NOD)-like receptor signaling pathway (Fig. 7A). A low abundance of *Pseudogulbenkiania* was related to cancer-related pathways, such as JAK-STAT signaling pathway, oocyte meiosis, phosphatidylinositol signaling system, prostate cancer, and Wnt signaling pathway (Fig. 7B). A low abundance of *Acidibacillus* was related to cytokine-cytokine receptor interaction. A high abundance of *Acidibacillus* was related to oocyte meiosis, phosphatidylinositol signaling system, TGF-β signaling pathway, and Wnt signaling pathway (Fig. 7C). A high abundance of *Succinimonas* was related to cell cycle, mismatch repair, oocyte meiosis, RNA degradation, and Wnt signaling pathway (Fig. 7D). The differences in pathway enrichment of IRIMs might explain the survival differences and different responses to immunotherapy among populations at different risks.

### The IRIMs were related to the response of chemotherapy drugs in BC

**Fig 8:**
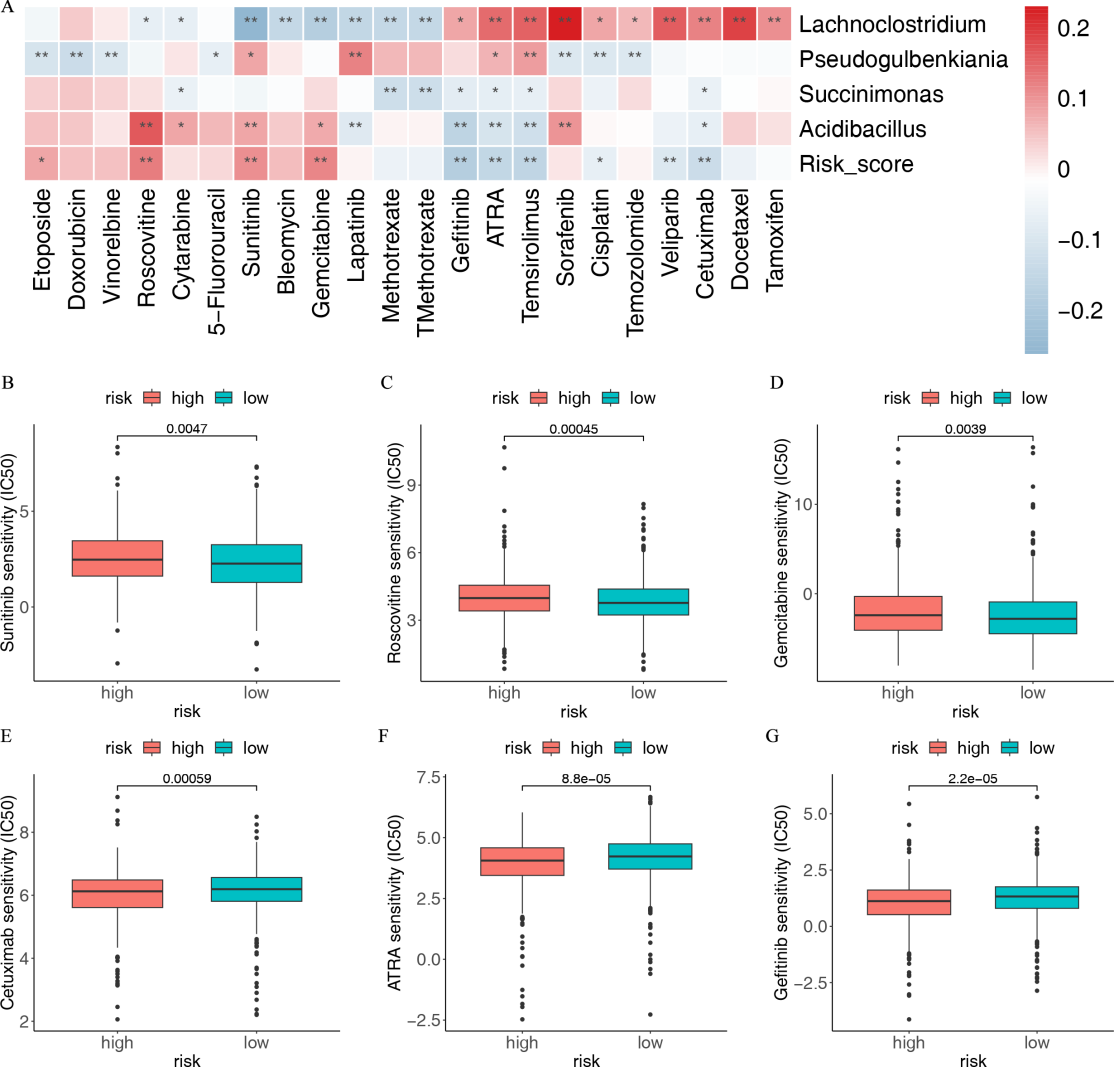
The sensitivity of chemotherapeutic agents between the two risk groups. (**A**) Correlation between the IC50 of the chemotherapeutic agents and immune-related microbial score. (**B–G**) Comparison of the IC50 values of chemotherapy and targeted agents in the two risk groups, including sunitinib, roscovitine, gemcitabine, cetuximab, ARTA, and gefitinib.

Chemotherapy is an essential means during cancer clinical treatment. Therefore, we explored the potential chemotherapy drugs for high-risk populations. The *Lachnoclostridium* was positively associated with the IC50 of tamoxifen, docetaxel, sorafenib, cetuximab, and veliparib but was negatively associated with the IC50 of sunitinib, methotrexate, bleomycin, and gemcitabine (Fig. 8A). The IMBPS score had positive associations with the IC50 of sunitinib, roscovitine, and gemcitabine but had negative associations with cetuximab, all-trans-retinoicacid (ARTA), gefitinib, and so forth (Fig. 8A). We further found that the two IMBPS groups differed in the sensitivity of common chemotherapeutic drugs (Fig. 8B). Patients with low risk contained a lower IC50 value of sunitinib, roscovitine, and gemcitabine, while high-risk patients contained a lower IC50 value of cetuximab, ARTA, and gefitinib. These results might guide the selection of chemotherapy drugs for high-risk patients and the development of microbial therapies to improve drug sensitivity.

## DISCUSSION

The development of immunotherapy has been one of the most significant advances in cancer treatment in the past decade (54). However, many patients did not respond to immunotherapy and developed resistance over time (55). Reshaping the TME has become a promising strategy for enhancing anti-tumor immune response (56). Recent evidence reveals that the intratumor microbiome could regulate the TME and local anti-tumor immunity (57, 58). However, the intratumor microbiomes that affect the immune microenvironment in BC have not been revealed sufficiently, which may play a revolutionary role in developing additional immunotherapies for BC.

The TIICs are closely related to immune therapy response (59, 60). In this study, we used ImmuCellAI to estimate the TME patterns in the BC patients. Two different immune subtypes of BC were identified, representing inflamed and non-inflamed tumors in immunology. The inflamed tumors with more anti-tumor TIICs have a better survival rate. Then, we associate immune subtypes with genetic, epigenetic, or intratumor microbiome characteristics to reveal their impact on the TME. The TMB was higher in the inflamed subtypes because tumor mutations may produce immunogenic new antigens (48). DNA methylation was higher in the non-inflamed subtypes. It has been confirmed that hypermethylation of the promoter of the new antigen leads to antigen loss in lung cancer patients (61). Functional analysis indicated that the two subtypes mainly differ in immune-related pathways and bacterial defense pathways. With LEfSe analysis, we identified 16 immune-related intratumor microbiomes based on two core immune subtypes of BC. Part of the IRIMs has been reported to be effective tumor prognosis biomarkers for cancer, such as *Lachnoclostridium* (62) and *Succinimonas* (63). In our study, the IRIMs-related prognosis model composed of four kinds of microorganisms (*Acidibacillus*, *Succinimonas*, *Lachnoclostridium*, and *Pseudogulbenkiania*) can predict the prognosis, TIME, and immunotherapeutic response of BC patients. The nomogram can further guide clinical workers in timely diagnosis and treatment.

Previous studies have shown that intratumor microbiomes can influence the abundance of TIICs by regulating immune-related genes (21, 24). We found that the *Flammeovirga, Lachnoclostridium, Terrabacter, Gelidibacter, Pseudogulbenkiania,* and *Nitrospira* were positively correlated with the anti-tumor immune cells. The IRIMs we found in BC were also reported that were correlated with immune components in other cancers. The *Flammeovirga, Lachnoclostridium,* and *Gelidibacter* can modulate chemokine levels and affect CD8+ T cell infiltration and were positive associations with infiltrating CD8+ T cells in cutaneous melanoma (24). We also found that *Lachnoclostridium* was positively correlated with immune regulator genes CD27 and TNFRSF4. CD27 co-stimulatory signaling could promote T cell activation and survival (64). The expression of TNFRSF4 promotes CD8+ and CD4+ T cell proliferation and survival (65). Therefore, *Lachnoclostridium* may affect the increase in CD8+ and CD4+ T cell infiltration levels by affecting CD27 and TNFRSF4. Furthermore, GSEA indicated that the four IRIMs were related to many immune- and cancer-related pathways to cause changes in signaling molecules and regulate the TME. The favorable *Lachnoclostridium* was associated with an activated natural killer cell-mediated cytotoxicity pathway. The unfavorable *Succinimonas* and *Acidibacillus* were associated with activated TGF-β and WNT/β-catenin signaling pathways, which could lead to the T cell exclusion and primary resistance to ICIs (66, 67). To sum up, the IRIMs might influence the immunotherapies by immune and signaling molecules in BC.

Multiple cancer treatment strategies based on intratumor microbiomes have been developed recently. The microbial-related therapy could exert therapeutic effect by coordinating chemotherapy (68–70), immunotherapy (71), and independent action to anticancer (72). The intratumor microbiome is crucial in reshaping the immune cancer immunotherapy response (73). The response of PD-1 blocking immunotherapy is related to the ratio of presumed favorable bacteria to unfavorable bacteria (74). The favorable intratumor *Lactobacillus reuteri* could release dietary tryptophan metabolite and facilitates immune checkpoint inhibitor treatment in melanoma (22). Ablation of unfavorable microbiomes can prevent tumor occurrence, reverse intratumor immune tolerance, and make immune checkpoint inhibitor work (57). Oral administration of *Bifidobacterium* can increase the activation of dendritic cells and improve tumor-specific CD8+ T cell response in mouse models (75). In clinical trials, supplementing live bacteria can improve the objective response rate and survival in patients with metastatic renal cell carcinoma treated with nivolumab-ipilimumab (76). Furthermore, independently of anti-PD-1, the Clostridiales can treat solid tumors currently resistant to immunotherapy by acting the potent anti-tumor effect via CD8+ T cells (72). Tumor resistance is the main issue that limits the efficacy of cancer chemotherapy drugs (77). More and more evidence indicate that microorganisms can affect the efficacy of cancer treatment (78). Intratumor bacteria could change the activity of chemotherapy drugs through the biotransformation of endogenous enzymes (68), regulating autophagy (69), and regulating host signaling pathways (70). Our study found that the *Flammeovirga, Lachnoclostridium, Terrabacter,* and *Gelidibacter* were related to an excellent response to immunotherapy. As to chemotherapy, we found that the IRIMs had close associations with chemotherapy drugs. Previous studies have shown how to regulate beneficial and harmful bacteria within tumors. The antibiotic-composed nanoparticles could treat tumors with bacteria infiltration by precisely killing pro-tumor bacteria efficiently (79). The engineered exogenous microbiota was also a promising cancer therapeutic strategy (80). Therefore, in the future, with the development of gene technology, modifying bacteria to enhance the anti-tumor activity is possible, and our study might provide a specific theoretical foundation.

However, some limitations should be discussed and analyzed further. Our study’s significant limitation is that it explored only a preliminary relationship between tumor microbiota and TME and prognosis, but a causal connection and specific mechanisms remain unknown. As another limitation, we generate results using the Kraken2 pipeline, which gets microbiome information from RNA sequencing. Some controversy about the standardization of pollutant treatment, batch effects, and machine learning methods in microbial analyses still exist in 2023 (81–83). Thus, it is necessary to carry out through experimental strategies such as microbial culture results and molecular biology validation.

### Conclusion

In conclusion, we identified two core immune cell infiltration patterns with different genetic, epigenetic, and intratumor microbiome factors in BC. The critical immune-related intratumor microbiomes were related to the prognosis, the abundance of TIICs, and the immunotherapeutic efficacy of BC. This study identified the potential intratumor microbiome, which might be the novel target to regulate immunotherapy in BC.

## Data Availability

The data and materials for all analyses are available at: https://portal.gdc.cancer.gov/repository, ftp://ftp.microbio.me/pub/cancer_microbiome_analysis/, and http://bioinfo.life.hust.edu.cn/ImmuCellAI#!/.

## SUPPLEMENTAL MATERIAL

Fig. S1 spectrum.04104-23-s0001.pdf Supplemental figure.

Table S1 to S8 spectrum.04104-23-s0002.xlsx Supplemental tables.

ASM does not own the copyrights to Supplemental Material that may be linked to, or accessed through, an article. The authors have granted ASM a non-exclusive, world-wide license to publish the Supplemental Material files. Please contact the corresponding author directly for reuse.
