# Supplementary material for: Multi-omics analysis elucidates the relationship between intratumor microbiome and host immune heterogeneity in breast cancer
Source: Microbiol Spectr. 2024 Mar 5;12(4):e04104-23. doi: 10.1128/spectrum.04104-23 (PMC10986513; doi:10.1128/spectrum.04104-23)
Supplement: Fig. S1 — Supplemental figure. [file spectrum.04104-23-s0001.pdf]

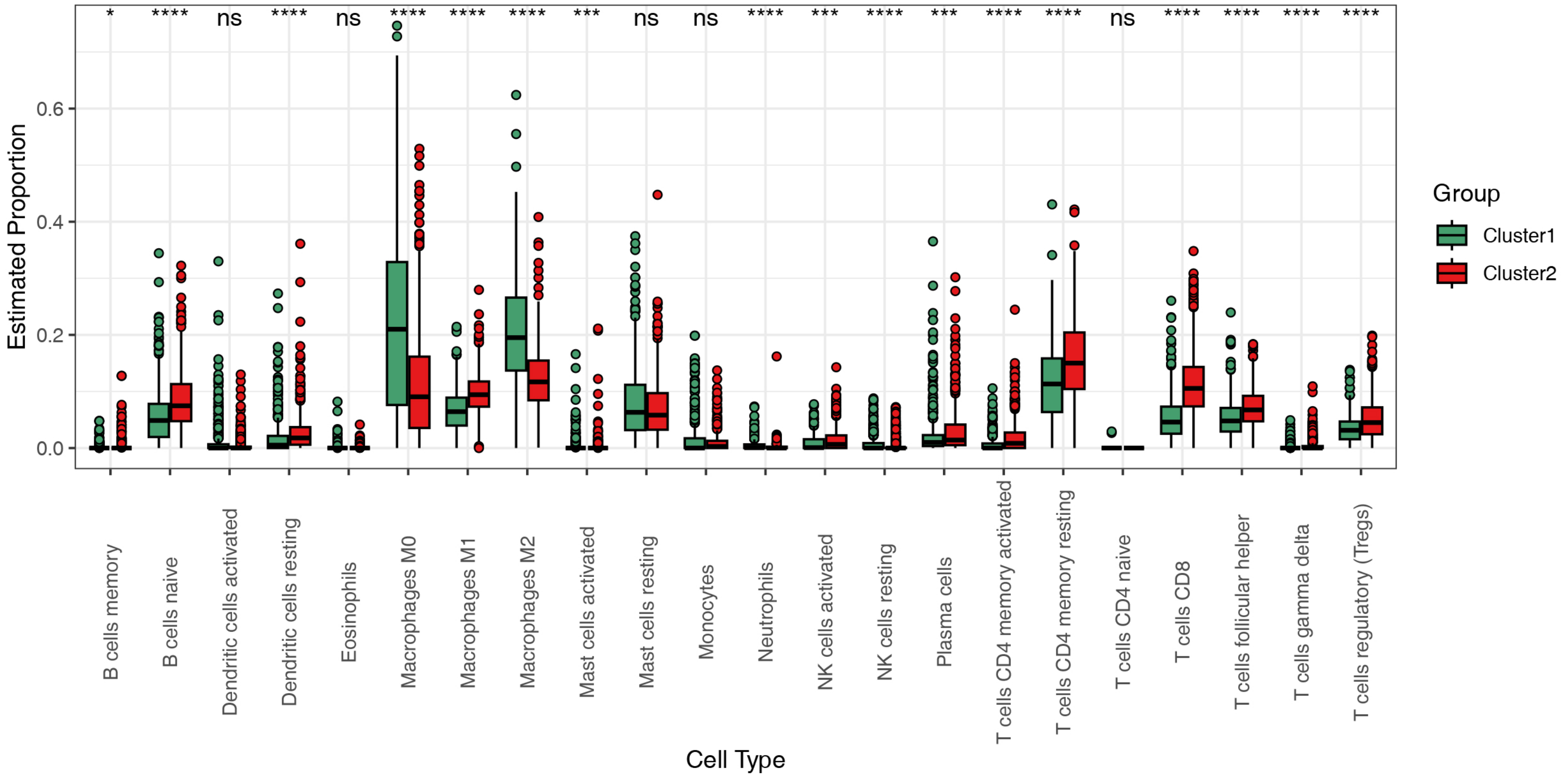

Supplementary Figure 1. Comparisons of the infiltration fractions of immune cells according to the CIBERSORTx between two clusters.
